# Chronic Treatment with Mood-Stabilizers Attenuates Abnormal Hyperlocomotion of GluA1-Subunit Deficient Mice

**DOI:** 10.1371/journal.pone.0100188

**Published:** 2014-06-16

**Authors:** Milica Maksimovic, Olga Y. Vekovischeva, Teemu Aitta-aho, Esa R. Korpi

**Affiliations:** 1 Institute of Biomedicine, Pharmacology, University of Helsinki, Helsinki, Finland; 2 Department of Pharmacology, Yong Loo Lin School of Medicine, National University Health System, Singapore; 3 Neurobiology and Ageing Programme, Life Sciences Institute, National University of Singapore, Singapore; 4 SINAPSE, Singapore Institute for Neurotechnology, Singapore; The Nathan Kline Institute, United States of America

## Abstract

Abnormal excitatory glutamate neurotransmission and plasticity have been implicated in schizophrenia and affective disorders. *Gria1−/−* mice lacking GluA1 subunit (encoded by *Gria1* gene) of AMPA-type glutamate receptor show robust novelty-induced hyperactivity, social deficits and heightened approach features, suggesting that they could be used to test for anti-manic activity of drugs. Here, we tested the efficacy of chronic treatment with established anti-manic drugs on behavioural properties of the *Gria1−/−* mice. The mice received standard mood stabilizers (lithium and valproate) and novel ones (topiramate and lamotrigine, used more as anticonvulsants) as supplements in rodent chow for at least 4 weeks. All drugs attenuated novelty-induced locomotor hyperactivity of the *Gria1−/−* mice, especially by promoting the habituation, while none of them attenuated 2-mg/kg amphetamine-induced hyperactivity as compared to control diet. Treatment with lithium and valproate reversed the elevated exploratory activity of *Gria1−/−* mice. Valproate treatment also reduced struggling behaviour in tail suspension test and restored reciprocally-initiated social contacts of *Gria1−/−* mice to the level shown by the wild-type *Gria1+/+* mice. *Gria1−/−* mice consumed slightly more sucrose during intermittent sucrose exposure than the wild-types, but ran similar distances on running wheels. These behaviours were not consistently affected by lithium and valproate in the *Gria1−/−* mice. The efficacy of various anti-manic drug treatments on novelty-induced hyperactivity suggests that the *Gria1−/−* mouse line can be utilized in screening for new therapeutics.

## Introduction

Abnormal major excitatory neurotransmission and neuroplasticity, driven by glutamatergic neurotransmitter system, have been implicated in schizophrenia and mood and anxiety disorders [1], [2]. Psychotic, cognitive and emotional disturbances are linked to hyperactive glutamatergic neurotransmission in the brain [3]. These disturbances can be reproduced in animals and human subjects by blockade of N-methyl-D-aspartate (NMDA) receptors, with a mechanism thought to involve enhanced non–NMDA receptor-mediated glutamate transmission [4], [5] and to be attenuated by agents inhibiting presynaptic glutamate release [6], [7]. Hyperglutamatergic state in the frontal cortical areas [8] and upregulated markers of excitotoxicity and neuroinflammation in the post-mortem frontal cortex [9] have been also observed in bipolar patients. Several susceptibility genes encoding for glutamate receptor subunits, including the *Gria1* gene encoding for GluA1 subunit of AMPA-type glutamate receptor (previously named GLU_A1_, GluR1, GluRA, GluR-A [10]), have been identified for bipolar disease [11], [12]. Most of them are overlapping with schizophrenia [13], [14], as these two illnesses share many behavioural characteristics.

An interesting finding has been the decreased GluA1 subunit expression in the post-mortem hippocampus, thalamus and frontal cortex of schizophrenic patients [15]–[19] and in the striatum of bipolar patients [20]. Mice lacking the GluA1 subunit [21] have been suggested to mimic some features of schizoaffective disorder and schizophrenia [22], [23]. These animals are abnormally active in the open field [21], [22], [24]–[26], but have a similar locomotor profile as wild-type animals in familiar home-cage environment [24]. Exaggerated exploration response provoked by a new object in the cage further point to abnormal reactivity to novel situations, although the *Gria1−/−* animals are known to habituate and recognize a familiar object [25]. Furthermore, abnormalities in working memory [21], [27], [28] and increased impulsive behaviour have been observed in the *Gria1−/−* mice [23]. These mice also exhibit a deficiency in pre-pulse inhibition and aberrant social interaction [25], [29], which resemble characteristic features seen in schizophrenic patients.

Here, we have focused on pharmacological features of the *Gria1−/−* mouse line, relevant for some positive symptoms of schizophrenia and/or mania, using a battery of behavioural tests to assess the predictive validity of the mouse model.

## Materials and Methods

### Ethics

All animal testing procedures were approved by the State Provincial Government of Southern Finland (ESAVI-0010026/041003/2010). All efforts were made to minimize the number and suffering of animals.

### Animals

*Gria1−/−* mice and their *Gria1+/+* wild-type (WT) controls were from heterozygous breeding, generated previously by inactivation of the *Gria1* gene [21] and genotyped as reported elsewhere [26]. The *Gria1−/−* mouse line is available at the Jackson Laboratory (B6N.129-*Gria1* ^tm2Rsp^/J, stock number: 019012). During experiments, mice were individually-housed or grouped-housed in same-sex cages under standard laboratory conditions (12-h light-dark cycle; lights on at 6∶00 A.M.; temperature 20–23°C; relative humidity 50–60%; aspen chip beddings).

### Drugs

The powdered laboratory chow (R36, Lantmännen Lantbruk, Stockholm, Sweden and RM1 (E) SQC FG, 811004, Special Diet Services, Essex, UK) was available *ad libitum* and it was mixed homogenously with drugs as follows: lithium carbonate (Sigma-Aldrich Corp St. Louis, MO USA) was added 1.2 g/kg chow for the first week and 2.4 g/kg until the end of treatment; sodium valproate (Deprakine, Sanofi Aventis Oy) 10 g/kg for the total treatment time; topiramate (Topiramat Ratiopharm) 27 mg/kg for the total treatment time; lamotrigine (Lamotrigin Ratiopharm) 75 mg/kg for the total treatment time. Doses aimed at human therapeutic levels and were based on the literature [30], [31] or pilot studies. Lithium and valproate group had free access to additional saline bottles to prevent possible ion imbalances during chronic treatments [32]. Control chow was made in the same way but without drugs. Animals were observed daily for any significant body weight changes or toxicity signs during the treatments.

### Drug Concentrations

Lithium and lamotrigine concentrations were analysed in hospital laboratories from blood samples of mice taken after the test of locomotor activity in novel environment. Trunk blood was collected by decapitation and serum separated by centrifugation. Concentration of lithium was determined by a colorimetric method [33] in NordLab Oulu (Oulu, Finland) and that of lamotrigine by a liquid chromatography after solid-phase extraction [34] in Rinnekoti-Foundation Laboratory (Espoo, Finland).

### Experimental Design

**Figure 1:**
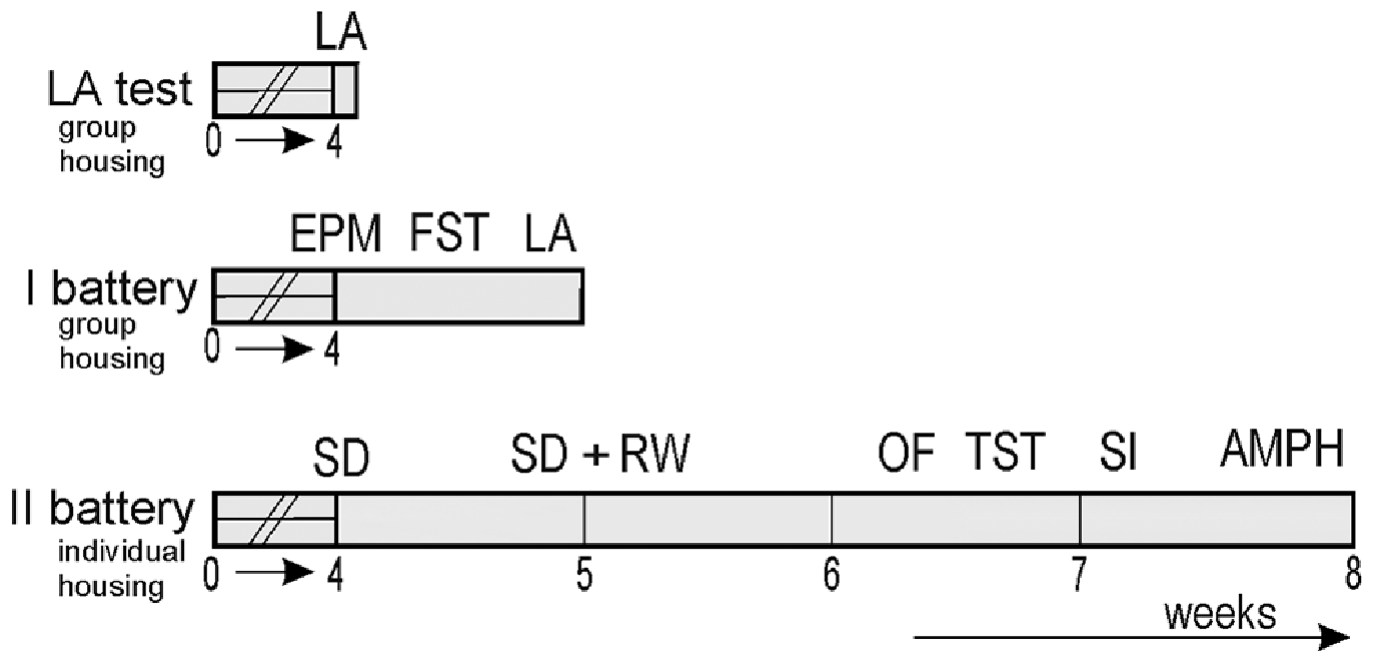
Experimental schedule for different cohorts of mice. LA, locomotor activity test; EPM, elevated plus maze test; FST, forced swimming test; SD, sucrose drinking; RW, running wheel access; OF, open field test; TST, tail suspension test; SI, social interaction test; AMPH, D-amphetamine-induced LA test.

Animals received drug treatments in their diet for 28 days, followed by behavioural testing while they still continued on the diets. Experimental design is presented in Fig. 1. We used test batteries to measure sets of specific behavioural features. The test order as well as a recovery break between the tests was designed to minimize the effect of previous test on subsequent ones. The first battery consisted of elevated-plus maze, forced swimming and locomotor activity tests in this order for two cohorts. In the second test battery, the animals were tested for sucrose preference using the two-bottle choice test to evaluate hedonistic behaviour towards sucrose, followed by open field test combined with new object installation, tail-suspension test, social interaction test and locomotor activity test after acute psychostimulant injection as the last test (the drug treatments lasted up to 2 months). Locomotor activity test was performed in several independent cohorts of WT and *Gria1−/−* mice and at the end of the first test battery and the results were pooled. All tests were performed between 08∶00 and 14∶00 h. In order to study sucrose drinking and wheel running, mice were separated to individual cages when the treatments began (4 weeks before the beginning of test) to minimize the effect of acute social isolation on hedonistic behavior [35] and this housing was kept throughout this battery of tests. The nesting material was provided in cages [36], [37]. Between the tests, the animals were handled at least two times weekly for body weight measurement or for changing to clean cages. Also, for certain time period the environment was enriched with running wheels to assess hedonistic behavior.

Locomotor activity (LA) in a novel environment was observed in plastic cages (40×30×20 cm) as described in detail [24]. Animals were habituated to the experimental room at least for 1 h before the test. Horizontal movements of eight to ten mice, placed in visually isolated cages in a sound-attenuated room at light intensity of 175 lx, were simultaneously recorded for 2 h using EthoVision Color-Pro 3.0 video tracking software (EthoVision System, Noldus Information Technology, Wageningen, Netherlands). All treatment groups are listed in Table 1.

**Table 1:** Characteristics of the treatment groups and drug concentrations.

Treatment	Gender	Genotype (numberof animals)	Age(weeks)	Initial bodyweight (g)	Final bodyweight (g)	Drug concentrationin blood
**Control**	Male	WT (16)	29.4±3.1	31.6±0.9	33.9±0.9***	/
		*Gria1−/−* (10)	30.1±2.2	29.1±0.9	31.6±0.9***	/
	Female	WT (11)	15.7±0.7	20.2±0.2	22.0±0.2***	/
		*Gria1−/−* (10)	22.3±4.0	21.1±1.0	23.7±0.9***	/
**Lithium**	Male	WT (8)	21.0±1.8	29.6±0.6	29.7±0.5	0.7±0.1
		*Gria1−/−* (7)	20.6±2.4	28.0±0.9	28.2±0.6	0.8±0.1
	Female	WT (13)	23.7±2.3	22.5±0.6	23.4±0.7	0.7±0.1
		*Gria1−/−* (12)	23.2±2.0	23.9±1.0	24.0±0.7	0.9±0.1
**Valproate**	Male	WT (13)	14.7±0.3	24.7±0.6	25.7±0.4	/
		*Gria1−/−* (10)	25.7±5.6	24.9±1.4	26.7±1.0	/
	Female	WT (4)	14.6±1.3	17.8±1.5	21.1±1.0	/
		*Gria1−/−* (4)	17.3±2.8	18.5±1.1	20.1±0.7	/
**Topiramate**	Male	WT (8)	16.7±0.7	28.0±1.0	30.1±1.2***	/
		*Gria1−/−* (8)	21.4±2.8	27.6±0.7	29.0±0.7**	/
	Female	WT (9)	17.9±0.7	22.4±0.8	23.9±0.7**	/
		*Gria1−/−* (10)	16.4±1.0	20.2±0.6	21.9±0.8**	/
**Lamotrigine**	Female	WT (8)	33.4±7.4	23.4±1.0	23.1±0.7	3.6±0.4**^#^**
		*Gria1−/−* (9)	33.8±4.1	25.1±1.0	24.3±0.7	5.0±0.4

Treatment groups, ages at the time of locomotor activity tests in novel cages, and body weights at the beginning and end of treatments are showed. Drug concentrations were analysed from serum samples collected after the locomotor activity tests and presented in mM (lithium) or µM (lamotrigine). Data are means ± SEM. *p<0.05, **p<0.01, ***p<0.001 compared to body weight at the beginning of treatments (paired *t*-test), ^#^ *p*<0.05 for the genotype difference (unpaired *t*-test).

Elevated plus maze (EPM) test was used to assess mouse anxiety [38]. The maze was made of grey plastic, elevated 50 cm from the floor level. It consisted of a central platform (5×5 cm), two open arms (5×40 cm with a 0.2 cm edge) and two enclosed arms (5×40×20 cm). The mice were placed individually on the central platform facing the open arm and allowed free exploration of the maze for 5 min. Central square was defined until 2 cm out of the central platform, allowing detection of the centre of animal. Movements were recorded and analysed automatically with the EthoVision software.

Forced swimming test (FST) and tail suspension test (TST) were done to assess animal’s coping with despair-like condition [39]. FST [24], [40], [41] was conducted so that each mouse was placed for 6 min individually in transparent cylindrical beakers (height 25 cm, diameter 15 cm) containing 3 l of water (23°C). In TST [24], [42], the mice were tape-attached individually by their tails on elevated metal bar for 6 min. Behavior of each mouse was video-recorded and analysed later by data acquisition program Ethograph (Ethograph software 2.06, RITEC, St. Petersburg, Russia) for the last 4 min of the tests. Behavioural analysis was focused on the mouse immobility which was indicated when the animal floated passively, making only small movements with the hind paws and/or tail to prevent sinking in the water during FST or when no struggling signs were obvious during TST.

Sucrose preference alone (SD) or in combination with running wheel (RW) was performed to assess hedonic propensity of the mice [43]. Voluntary sucrose drinking [8% weight/volume (w/v)] was evaluated using short-term intermittent protocol [44], [45]: on alternate days the mice obtained access to choose between water and sucrose. The alternation of sucrose-free and sucrose days (S1, S2 and S3) was repeated 3 times, and thereafter the alternation was repeated 3 times (S4, S5 and S6) with running wheels (RW; ENV-044 model; Med Associates, Inc., St. Albans, Vermont, USA). The position of water and sucrose bottle was switched pseudo-randomly to prevent the development of place preference towards the sides. Fluid intake and body weights were monitored daily. Sucrose solution intake (in ml) was calculated by dividing loss of sucrose bottle weight with 1.08 [weight in grams of 1 ml of 8% (w/v) sucrose solution]. Sucrose preference (%) was calculated as a percentage of sucrose intake out of the total fluid intake (sucrose plus water).

Open field (OF) test combined with new object exploration was performed to evaluate anxiety and mouse explorative activity [46]. The animals were placed individually on the centre of empty cage (33×55×19 cm) divided into fifteen squares (11×11 cm) initially for 3 min. Testing was performed at the light intensity of 175 lx. Then, a round, textured object (diameter = 4 cm) with three 1-cm holes was placed on the centre for the next 3 min [41]. Mouse behaviour was video-recorded and analysed later by Ethograph for the last 3 min of the test. Arena of the OF was divided into peripheral and central zones in the video tracking software (EthoVision), which was used to track the mouse automatically for the whole 6 min duration of the test. Object-related behaviours (sniffing, manipulation and nose-pokes of the object) were counted as total object interactions. Other behaviours including the rearing, locomotion and individual behaviours (any other behavior which does not include locomotion) were divided to central and peripheral behaviours according to the virtual zones of the OF.

Social interaction (SI) was evaluated for 10 min on a new territory among animals receiving the same treatment [41]. Two or three animals were simultaneously introduced to a novel cage with fresh bedding. The behaviour of animals was video-recorded. Behavior of every mouse in the temporarily-formed group was analysed by the Ethograph software for (1) individual behavior without contacts with other members, such as locomotor activity, (2) reciprocal (simultaneously-initiated) contacts, and (3) passive contacts initiated by other group members. All observed contacts were non-aggressive, although aggressive behaviour was expected.

Response to psychostimulants was tested 30 min after a single i.p injection of 2 mg/kg amphetamine diluted in saline (D-amphetamine sulphate, Dexedrine, GlaxoSmithKline, Brentford, UK) in a volume of 10 ml/kg. Mouse locomotion was monitored for 1 h in a novel arena by using the EthoVision system and software.

### Statistics

Statistics were carried out using PASW Statistic 18 software (SPSS Inc., Chicago, IL, USA). Multivariate ANOVA (two-way) followed by a Bonferroni (*p*<0.05) post hoc test was used to analyse the data obtained from EPM, FST, TST, new object exploration and social activity tests. For the repeated measurements such as locomotor activity and sucrose consumption, analysis for repeated measurements followed by a Bonferroni post hoc test (*p*<0.05) was applied. Kaplan-Meier survival analysis with a Mantel-Cox non-parametric test (*p*<0.05) was used to analyse the latencies to immobility, to the contact with the new object and to the social contact. Spearman’s correlation coefficient was used to test correlation between sucrose drinking and activity on running wheels. Two-tailed *t*-test was used to compare differences in results for body weights (paired), drug concentrations (unpaired) and LA (unpaired) obtained in different cohorts. All behavioural elements or group of the elements obtained by the Ethograph software were statistically analysed in four measurements: total duration (sum of the duration of the element during the test), medial duration (ratio of the element duration to its total frequency), total frequency, and relative frequency (ratio of the element frequency to the sum of all frequencies of the observed elements).

## Results

### Chronic Treatment with Mood Stabilizers Lithium and Valproate and Effects on Hyperactivity of *Gria1−/−* Mice

As locomotor activity tests were performed in several cohorts with different experimental schedules (as an independent test and at the end of the first battery, Fig. 1), we analysed statistically whether the previous tests affected the later ones, by using *t*-tests within each 6 experimental groups. No difference was observed in the total 2-h locomotor activity within the control-treated *Gria1−/−* mice (t_18_ = 0.02, *p>*0.05) between the separate test [571±45 m (10), mean ± SEM, (n)] and the last test of the first battery [572±42 m (10)], nor within the corresponding groups of control-treated WT mice [t_25_ = 0.25, *p*>0.05, 308±25 m (12) and 300±22 m (15)]. Nor were there any differences in LA between independently performed and after EPM and FST performed tests within the lithium-treated *Gria1−/−* mice [t_17_ = 1.47, *p*>0.05, 417±51 m (7) and 509±38 m (12)], lithium-treated WT mice [t_19_ = 0.47, *p*>0.05, 281±23 m (8) and 302±31 m (13)], valproate-treated *Gria1−/−* mice [t_12_ = 1.17, *p*>0.05, 426±24 m (7) and 375±36 m (7)] nor valproate-treated WT mice [t_15_ = 0.33, *p*>0.05, 240±18 m (7) and 229±25 m (10)]. We conclude that previous testing experience did not influence the novelty-induced LA.

**Figure 2:**
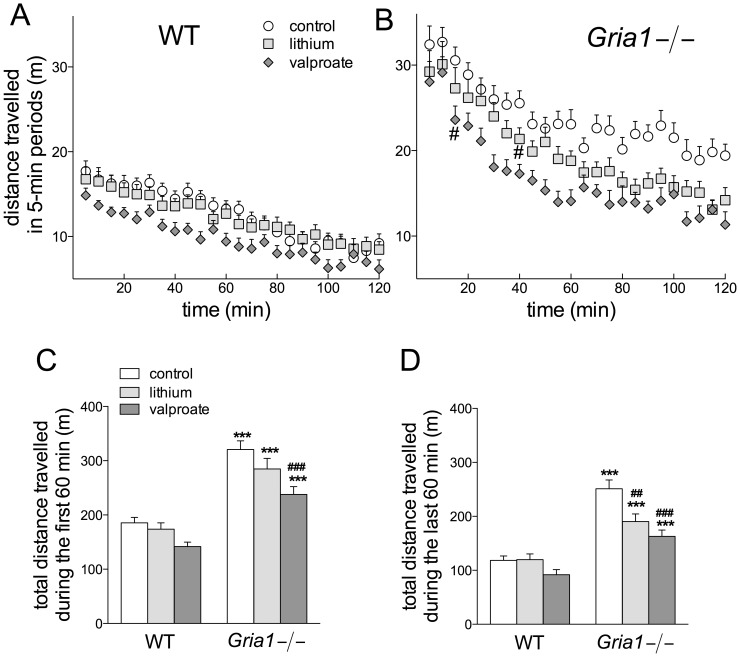
Effects of chronic lithium and valproate on hyperactivity of the *Gria1−/−* mice. Locomotor activities of WT and *Gria1−/−* mice treated chronically with control diet and lithium- and valproate-supplemented diets in 5-min time intervals for the whole 2-h period (**A, B**) and cumulative activities during the first and last 60 min of exposures to novel arena (**C, D**). Data are means ± SEM (n = 14–27). ***p<0.001 for the differences between genotypes after the same treatment; ^##^ *p*<0.01, ^###^ *p*<0.001 for the differences from controls of the same genotype (two-way ANOVA followed by Bonferroni *post-hoc* test). In panel **B**, the earliest significant reduction from the control activity has been marked by ^#^(*p*<0.05).

In the first 60-min of LA test, there was a significant genotype × treatment × gender interaction (F_2,106_ = 3.20, *p*<0.05) after three-way ANOVA. Bonferroni post-hoc comparisons showed that female and male *Gria1−/−* mice travelled longer distances than WT mice, in all treatment groups (*p*<0.05). There were no significant gender differences in LA scores within *Gria1−/−* mice on control-, lithium- or valproate-diets (*p*>0.05), nor within the WT mice on these diets (*p*>0.05). Both lithium (*p*<0.05) and valproate (*p*<0.05) treatments were efficient in reducing locomotor hyperactivity of female *Gria1−/−* mice compared to control treatment. The drugs did not affect LA of WT mice in comparison to control diet. Valproate-treated *Gria1−/−* (*p* = 0.075) and WT (*p* = 0.064) male mice tended to show reduced LA compared to respective control-treated mice. Thus, only a slight difference in response of female and male *Gria1−/−* mice to the lithium treatment appeared during the first 60-min of the test. Statistical analysis showed that gender did not interact with other factors in the second 60-min of exposure to novel cages, when the effect of the drugs on the locomotor activity was predominant (Fig. 2). Because of that, we analyzed below pooled female and male data also for the first 60-min of the test using two-way ANOVA and these results are presented on Fig. 2C.

Locomotor activity of *Gria1−/−* mice during the first 60-min exposures to novel environment after the chronic drug treatments was markedly increased as compared to that of WT mice (F_1,112_ = 101.17, *p*<0.001) (Fig. 2A–C). Only valproate-treated *Gria1−/−* had reduced LA as compared to control *Gria1−/−* mice (F_2,112_ = 10.18, *p*<0.001). The LA of the *Gria1−/−* mice continued to be higher than that of WT mice till the end of the 2-h monitoring period, but cumulative LA during the last 60 min showed that both lithium and valproate were efficient in reducing LA in *Gria1−/−* mice (F_2,112_ = 4.82, *p*<0.05 for genotype × treatment interaction), but not in WT animals (Fig. 2D). Thus, chronic treatments led to increased habituation of the *Gria1−/−* mice. Indeed, the effects were observed only after 15 min by valproate and after 40 min by lithium (Fig. 2B), and the reductions were more pronounced for 60–120 min than 0–60 min periods (Fig. 2C and D). The differences between treatments were indistinguishable after 70 min (Fig. 2B).

### Effects on Elevated Plus-maze Test and Open Field Exploration

**Figure 3:**
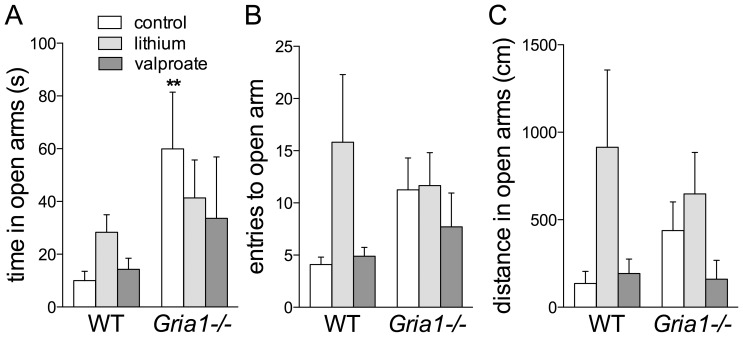
Effects of chronic drug treatments on behaviour of *Gria1−/−* mice in elevated-maze test of anxiety. Control *Gria1−/−* spent more time in the open arms than control WT mice. Chronic lithium and valproate did not affect the time spent in (**A**) or entries to (**B**) or distances travelled in open arms (**C**). Data are means ± SEM (n = 7–12). **p<0.01 for the difference between genotypes after the same treatment (two-way ANOVA followed by Bonferroni *post-hoc* test).

*Gria1−/−* mice visited the centre of the elevated plus-maze more frequently than the WT mice (F_1,54_ = 5.86, *p*<0.01) and spent less time in the closed arms (F_1,54_ = 16.10, *p*<0.001) (data not shown), which measures were not affected by drug treatments. Control *Gria1−/−* mice spent more time in the open arms than the control WT mice (F_1,54_ = 5.20, *p*<0.05) independent of the treatments (Fig. 3A). No genotype or treatment effects were observed on number of entries to open arms or on distance travelled in the open arms (Fig. 3B–C).

**Figure 4:**
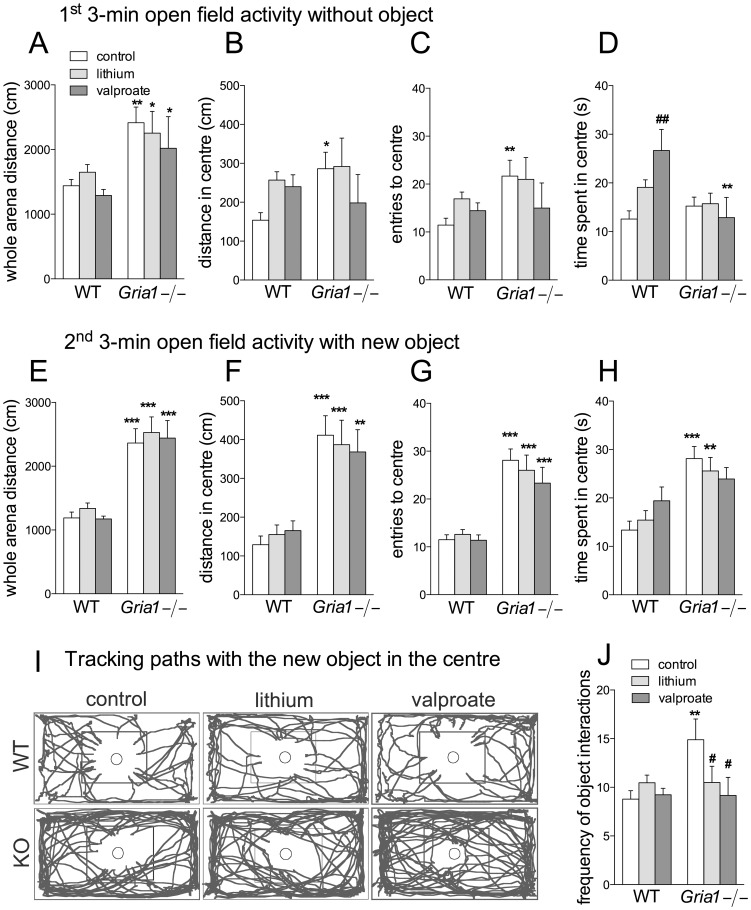
Effects of chronic drug treatments on open field activity and interaction with a novel object. Distance travelled in the whole arena (**A, E**) and in the centre (**B, F**), entries to the centre (**C, G**) and time spent in the centre (**D, H**) during the first 3 min before the object was introduced (**A**–**D**) and during the next 3 min when a novel object was located in the centre of arena (**E**–**H**). (**I**) Representative tracking paths for *Gria1−/−* and WT mice during the 2^nd^ 3-min period with an object, with a square in the centre delineating visual centre of arena. (**J**) Frequencies of object interactions, scored using Ethograph software. Data are means ± SEM (n = 6–15). *p<0.05, **p<0.01, ***p<0.001 for the differences between genotypes after the same treatment; ^#^ *p*<0.05, ^##^ *p*<0.01 for the differences from the control within the same genotype (two-way ANOVA followed by Bonferroni *post-hoc* test).

*Gria1−/−* mice moved more than WT mice in the whole open field arena during 3 min before the object was introduced (Fig. 4A; F_1,61_ = 19.94, *p*<0.001), and chronic treatments with lithium and valproate did not affect total movements (F_2,61_ = 1.10, *p*>0.05). *Gria1−/−* mice visited the OF centre more frequently (Fig. 4C; F_1,61_ = 4.85, *p*<0.05) than WT mice, but travelled similar distances in the centre as them (Fig. 4B; F_1,61_ = 1.51, *p*>0.05). Valproate increased the time spent in the central zone in WT mice, but not in *Gria1−/−* mice (Fig. 4D; genotype × treatment interaction F_2,61_ = 3.78, *p*<0.05).

After the object was introduced in the OF for the next 3 min, *Gria1−/−* mice kept moving more in the whole arena (Fig. 4E; F_1,61_ = 92.90, *p*<0.001) as well as in the arena centre (Fig. 4F; F_1,61_ = 55.23, *p*<0.001), independently of chronic treatments. *Gria1−/−* mice visited the central zone with the object more often (Fig. 4G; F_1,61_ = 81.15, *p*<0.001) and stayed there longer (Fig. 4H; F_1,61_ = 22.32, *p*<0.001). Control *Gria1−/−* mice were in contact with the object more frequently than WT mice, while treatments of the *Gria1−/−* mice with valproate and lithium decreased the frequency to the level of the corresponding WT mice (Fig. 4J; F_2,61_ = 3.97, *p*<0.05).

### Effects on Tests for Goal-directed Behaviours

**Figure 5:**
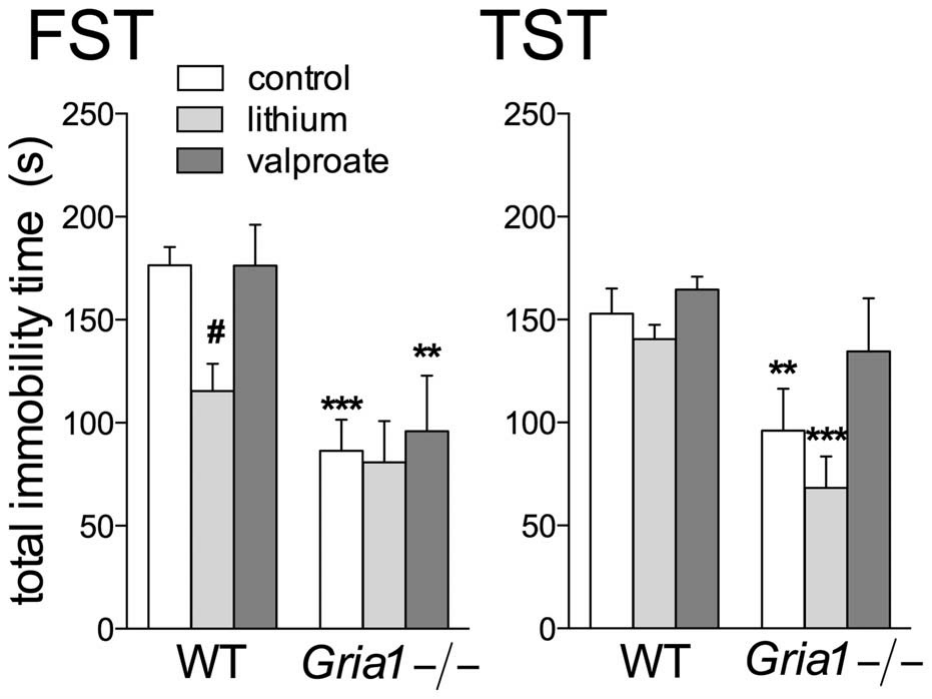
Effects of chronic drug treatments in behavioural despair paradigms. *Gria1−/−* mice were less immobile than the WT mice in both the forced swimming (FST) and tail suspension (TST) tests. Data are means ± SEM (n = 7–13). **p<0.01, ***p<0.001 for the differences between genotypes after the same treatment; ^#^ *p*<0.05 for the differences from the control within the same genotype (two-way ANOVA followed by Bonferroni *post-hoc* test).

Forced swimming test (FST) has been validated to examine increased vigour and goal-directed behavioural pattern of mania [39]. The increased goal-directed behaviour in both FST and tail suspension test (TST) has been already reported in *Gria1−/−* mice [24]. In the FST, *Gria1−/−* mice were less immobile as compared to the WT mice (Fig. 5; F_1,60_ = 23.87, *p*<0.001), especially the control and valproate-treated groups. Lithium-treated WT mice showed a trend towards being less immobile than other WT groups (F_2,60_ = 3.13, *p* = 0.051). Kaplan-Meier analysis showed that immobility was observed later in the control and lithium-treated *Gria1−/−* mice than in the corresponding WT mice (14.15, *p*<0.0001 and 5.73, *p* = 0.017, respectively, data not shown).

In the TST, again the *Gria1−/−* mice were less immobile than the WT mice (Fig. 5; F_1,61_ = 22.79, *p*<0.001), especially the control and lithium-treated *Gria1−/−* mice. Valproate prolonged the immobility in the *Gria1−/−* mice (F_2,61_ = 5.34, *p*<0.01). Kaplan-Meier analysis showed that lithium-treated *Gria1−/−* mice demonstrated immobility later than control *Gria1−/−* mice (log-rank Mantel-Cox 14.32, *p*<0.0001) and lithium-treated WT mice (13.50, *p*<0.0001) (data not shown).

We compared the immobility times in FST and TST tests that were carried out in group- or individually-housed mice, respectively. Within the *Gria1−/−* mice, no difference (t_18_ = 0.39, *p*>0.05) was observed in the total immobility time of between FST and TST [86.4±15.1 s (12), mean ± SEM, (n) and 96.1±20.3 s (9), respectively], and similarly, the immobility times of WT mice in FST [176.5±8.8 s (12)] and TST [152.9±12.2 s (14)] were identical (t_24_ = 1.52, *p*>0.05). Also, lithium- (t_20_ = 0.46, *p*>0.05) and valproate-treated (t_11_ = 1.03, *p*>0.05) *Gria1−/−* mice spent similar times immobile in these tests, as did lithium- (t_26_ = 1.75, *p*>0.05) and valproate-treated (t_21_ = 0.63, *p*>0.05) WT mice. It seems that the housing conditions (individual or group-housing) hardly affected the behavior of *Gria1−/−* and WT mice.

### Effects on Social Interaction

**Figure 6:**
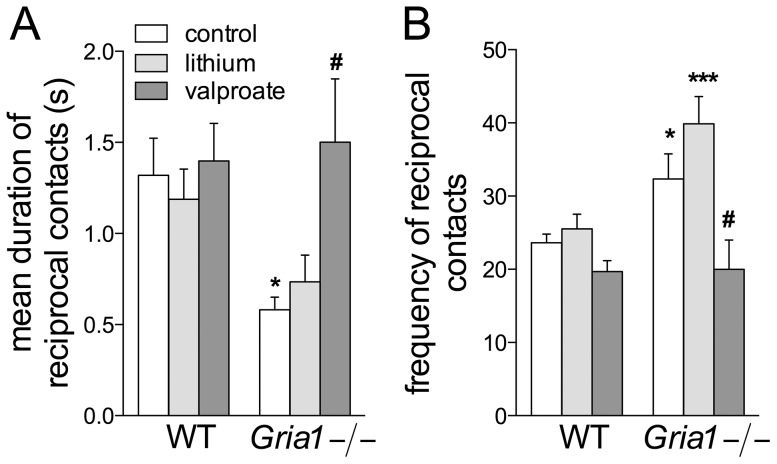
Effects of chronic drug treatments on social activity on a new territory. Two or three animals on the same treatment were observed for 10(simultaneously-initiated) contacts. The contacts were shorter (**A**) but more frequent (**B**) among the control *Gria1−/−* mice than among the WT mice. Chronic valproate, but not lithium, moderated the activity of the *Gria1−/−* mice to the level found in the WT mice. Data are means ± SEM (n = 6–15). *p<0.05, ***p<0.001 for the differences between genotypes after the same treatment; ^#^ *p*<0.05 for the differences from the control within the same genotype (two-way ANOVA followed by Bonferroni *post-hoc* test).

Reciprocally initiated contacts were shorter-lasting but more frequent in *Gria1−/−* mice than in WT mice (F_2,60_ = 4.64, *p*<0.05), while valproate treatment levelled the *Gria1−/−* mice behaviour to that of the WT mice (Fig. 6AB; treatment effect F_2,60_ = 3.46, *p*<0.05 and F_2,60_ = 3.76, *p*<0.05 for the mean time and frequency, respectively). Unlike WT, *Gria1−/−* mice spent more time in passive interaction receiving contacts from other mice (F_1,60_ = 4.24, *p*<0.05; data not shown), independently of the treatments with lithium and valproate. They were engaged more frequently in individual behaviour than WT mice (F_1,60_ = 31.22, *p*<0.001) and lithium increased it over the control and valproate-induced levels (F_2,60_ = 9.91, *p*<0.001; data not shown). Lithium delayed the appearance of reciprocal contacts between the group members in WT mice (log-rank Mantel-Cox 5.11, *p*<0.05), and reciprocal contacts were observed later in control *Gria1−/−* than WT mice (log-rank Mantel-Cox 4.70, *p*<0.05; data not shown).

### Effects on Amphetamine-induced Hyperactivity

*Gria1−/−* and WT mice are similarly activated by acute amphetamine challenge [26]. Here, we studied whether this dopaminergic challenge would be affected by chronic drug treatments. *Gria1−/−* mice preserved their higher locomotor activity after 2 mg/kg amphetamine challenge throughout the 60-min monitoring period as compared to WT mice, independently of the chronic treatments with lithium and valproate (F_11,671_ = 12.37, *p*<0.001 and F_1,61_ = 50.29, *p*<0.001 for time intervals and genotype, respectively). Locomotor activity distances (in meters as estimated by using Ethovision video-tracking) were for the control, lithium and valproate groups of the WT mice: 311±18 (mean ± SEM, n = 14), 342±22 (15) and 295±11 (13), respectively, and for the corresponding treatment groups of the *Gria1−/−* mice: 507±59 (9), 489±32 (10) and 462±24 (6), respectively.

### Effects on Sucrose Preference and Activity on Running Wheels

**Figure 7:**
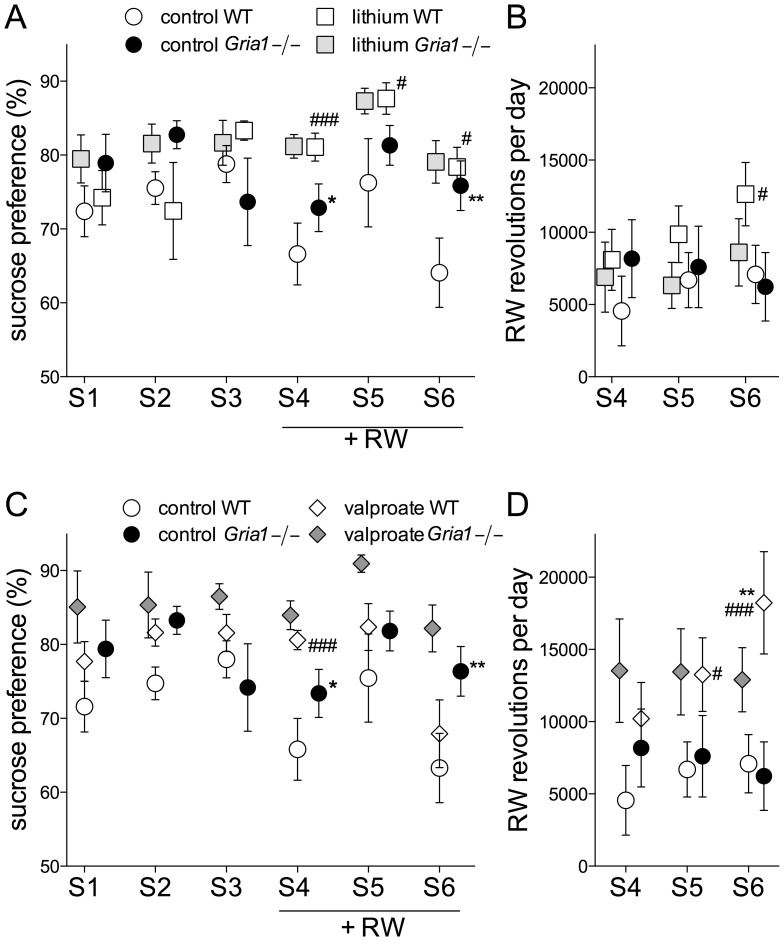
Effects of chronic lithium and valproate on sucrose preference and running wheel activity *Gria1−/−* mice. Preference for sucrose-containing solution over plain water, with and without an access to running wheels (RW) on sucrose-choice days (S1, S2 and S3) in animals treated with lithium (**A**) and valproate (**C**). Running wheel activity during the sucrose-choice days (S1, S2, S3) in animals treated with lithium (**B**) and valproate (**D**). Data are means ± SEM (n = 6–14). *p<0.05, **p<0.01 for the differences between genotypes after the same treatment; ^#^ *p*<0.05, ^###^ *p*<0.001 for the differences from the control treatment within the same genotype (two-way ANOVA followed by Bonferroni *post-hoc* test).

To compare the mouse lines and to assess the effects of lithium and valproate on hedonic behaviour, we tested the *Gria1−/−* and WT mice for preference of sucrose-containing solution and for running wheel activity. The *Gria1−/−* mice had higher preference for sweet taste than WT mice (F_1,57_ = 8.87, p<0.01), although all animals preferred sucrose solution over plain water. Animals receiving lithium (Fig. 7A) and valproate (Fig. 7C) increased sucrose consumption as compared with respective control animals (F_2,57_ = 8.86, *p*<0.001). Interestingly, the control WT mice reduced their consumption of sucrose during the first (S4) and third (S6) session in the presence of running wheels (F_5,285_ = 5.12, *p*<0.01). Otherwise the access to running wheels little affected the preference for sucrose, although particularly the valproate-treated WT mice clearly increased their activity on running wheel on the last session (S6, Fig. 7D; F_2,250_ = 5.83, *p*<0.01). Also lithium treatment increased running activity in the WT mice on session S6 (Fig. 7B). Thus, no correlation (*p*>0.05) was observed between running wheel activity and sucrose preference in WT (0.01) or *Gria1−/−* mice (0.091).

### Effects of Anticonvulsants Topiramate and Lamotrigine on Behavior of *Gria1−/−* Mice

**Figure 8:**
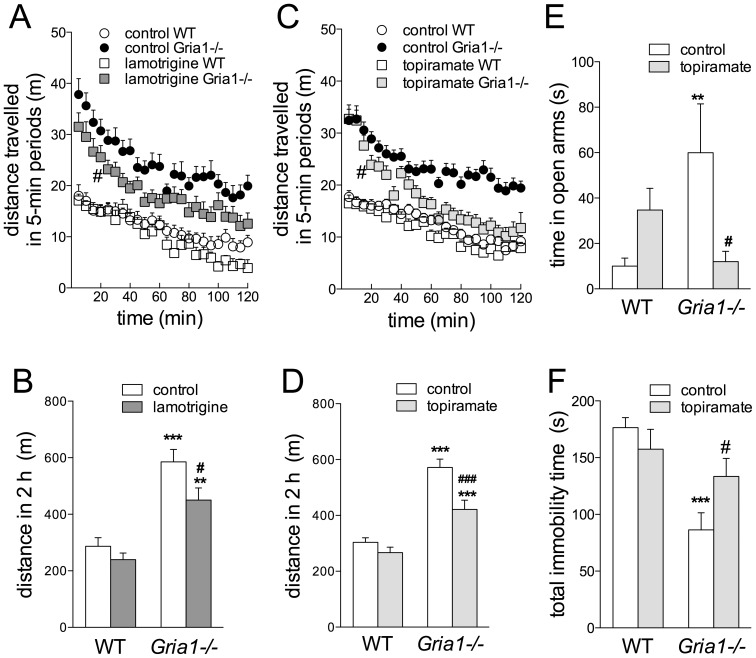
Effects of chronic treatments with anticonvulsants lamotrigine and topiramate on behavior of *Gria1−/−* mice. Cumulative locomotor activities of *Gria1−/−* and WT mice treated chronically with lamotrigine- or topiramate-supplemented chow for 5-min time intervals (**A, C**) and for the whole 2-h experiment (**B, D**). Data are means ± SEM (n = 8–27). In **A** and **C**, the earliest significant point from the control activity has been marked by ^#^(*p*<0.05). Topiramate treatment reduced the time spent in open arms of elevated-plus maze (**E**; means ± SEM, n = 9–12) and increased immobility time in forced swimming test (**F**; means ± SEM, n = 11–12) in *Gria1−/−* mice. **p<0.01, ***p<0.001 for the difference between genotypes after the same treatment; ^#^ *p*<0.05, ^###^ *p*<0.001 for the difference from the control within the same genotype (two-way ANOVA followed by Bonferroni *post-hoc* test).

In WT and *Gria1−/−* females, lamotrigine reduced cumulative 2-h novelty-induced LA in *Gria1−/−* mice (Fig. 8A–B; treatment effect F_1,35_ = 5.93, *p*<0.05, genotype effect F_1,35_ = 46.38, *p*<0.001), with the earliest significant reduction taking place at 20 min after starting the test (Fig. 8A).

There was no difference (t_16_ = 1.58, *p*>0.05) in LA of topiramate-treated *Gria1−/−* mice between the two LA tests [performed independently (351±31 m (6), mean ± SEM (n)) or after other tests in the battery (457±44 m (12)], nor within the WT mice [t_15_ = 0.15, *p*>0.05; 271±12 m (6) *vs.* 265±30 m (11)]. Therefore, the data were pooled, analysed and presented in Fig. 7C–D. The gender effect was not significant for either two 1-h time intervals (F_1,74_>0.01, *p*>0.05, three-way ANOVA) and, therefore, female and male data were pooled. Chronic treatment with topiramate reduced 2-h novelty-induced hyperactivity specifically in *Gria1−/−* mice in a time-dependent manner, without affecting WT animals (Fig. 8C–D; F_23,1794_ = 3.09, *p*<0.01 for time interval × genotype × treatment interaction). The first effect of topiramate on locomotor activity was observed after 20 min (Fig. 8C). In the EPM, treatment with topiramate reduced the time that *Gria1−/−* mice stayed on the open arms (Fig. 8E; F_1,39_ = 7.37, *p*<0.05 for genotype × treatment interaction), suggesting anxiogenic or reduced risk-taking effects. Topiramate increased total immobility time in *Gria1−/−* mice, but not in WT mice (Fig. 8F, F_1,43_ = 5.16, *p*<0.05 for genotype × treatment interaction) in the FST. Kaplan-Meier analysis showed that topiramate-treated *Gria1−/−* mice demonstrated immobility earlier than the control *Gria1−/−* mice (log-rank Mantel-Cox 4.26, *p* = 0.039).

## Discussion

In this study, we confirmed the elevated activity and highly exploratory phenotype of *Gria1−/−* animals and conducted a predictive assessment of efficacy of chronic treatments of standard and novel, structurally and mechanistically different mood stabilizers on a battery of behavioural tests for hyperactive aspects of disorders, such as bipolar mania-like illness, schizophrenia and schizoaffective disorder. We found that the treatments with lithium, valproate, topiramate and lamotrigine all reduced hyperactivity by increasing habituation of the *Gria1−/−* animals in a novel environment and variably affected other behaviours.

Locomotor over-activity and increased exploration in open space as well as over-reactivity to new objects were characteristic for the *Gria1−/−* mice, indicative of high novelty seeking and risk-taking behaviour. This behaviour was particularly sensitive to both lithium and valproate. Dysfunctional exploration pattern has been observed in bipolar manic and schizophrenic patients [47], [48]. Interestingly, bipolar manic patients are more mobile and habituate faster than schizophrenic patients. Moreover, bipolar patients are more interested in new objects and spend more time near to the object [47], [49]. Thus, specific behavioural features differentiate bipolar patients from schizophrenic ones and could be used as discriminating criteria between these disorders. Moreover, behavioural disinhibition pattern was suggested as endophenotype of bipolar disorder [50]. In the present work, chronic treatment with the mood stabilizers studied here attenuated the 2^nd^-h hyperlocomotion in a novel environment in the *Gria1−/−* mice, but were not effective in reducing the initial hyperactivity or the acutely exacerbated locomotion by amphetamine challenge. *Gria1−/−* mice show normal home-cage activity pattern and diurnal rhythm [24] and amphetamine elevates the activity similarly as is the WT mice [26], which suggest that their abnormal novelty-induced hyperactivity resembles more mania-type behaviour than attention deficit hyperactivity disorder-type of behaviour.

Novelty-induced hyperactivity of the *Gria1−/−* mice is greatly reduced by acute blockade of AMPA receptors with NBQX [24] and by mGlu2/3 receptor agonist LY354740 [51], which results are consistent with hyperactive glutamate system in the *Gria1−/−* mice. Our recent c-Fos mapping data suggest overactivity of the dorsal hippocampus of the *Gria1−/−* animals in a novel situation [24], [51]. Of the mood stabilizers studied here, topiramate and lamotrigine reduce glutamate functions by inhibiting glutamate receptors and glutamate release, respectively [52]–[54], which could explain their efficacy. Also treatment with lithium is known to indirectly affect NMDA-type glutamate receptor function, subunit expression and phosphorylation and activation of the related intracellular signalling cascades, such as phospholipase PLA_2_ and nitric oxide (NO) pathways [55]–[58], while the NMDA receptor antagonists potentiate the actions of lithium [59]. However, other mechanisms than glutamate antagonism are likely to be involved also, since valproate is not known to antagonize the glutamate system, and indeed valproate has failed to protect from NMDA-induced seizures [60]. Protein kinase C and extracellular signal-regulated kinase cascades constitute as shared targets for lithium and valproate and are involved in mediating their anti-manic actions on various facets of the disease [61]. These pathways may also be among the mediators of the anti-hyperactive effect of lithium and valproate in the *Gria1−/−* model of abnormal hyperactivity.

Chronic treatment with lithium could also suppress hyperlocomotion via presynaptic mechanisms that decrease release of catecholamines or inhibit their synthesis [62]. Indeed, dopamine D2 receptor antagonist haloperidol somewhat reduces hyperactivity in the *Gria1−/−* and WT mice [25]. However, depletion of dopamine levels by inhibition of tyrosine hydroxylase did not affect the locomotor phenotype of these mice [22], neither was there any differential activation of the ventral tegmental area dopaminergic (VTA DA) neurons or striatal neurons between *Gria1−/−* and WT mice after 2 h in novel environment [24], and therefore, dopaminergic mechanisms are unlikely to decisively contribute to the hyperlocomotor phenotype. On the other hand, glutamate receptor neuroplasticity in VTA DA neurons is associated with the effects of rewarding drugs of abuse [63], [64], and this neuroplasticity process might be deficient in *Gria1−/−* mice as the opioid morphine failed to induce an increase of AMPA/NMDA ratio of VTA DA neurons in *Gria1−/−* mice like it did in WT animals [65]. State-dependent place conditioning with morphine is abnormal in *Gria1−/−* mice [65]. In the present study, of the two naturally rewarding stimuli, sucrose drinking and running wheel activity, only sucrose drinking test was useful to discriminate between the *Gria1−/−* and WT mice, while running activity did not differ between the genotypes. Dopaminergic projection from the VTA is important for appetitive behaviour and hedonic responses to palatable food [66], with a lesion of this projection decreasing sucrose intake [67]. Activation of VTA DA neurons by disinhibition via cannabinoid CB_1_ receptor-dependent mechanism has been linked to rewarding properties of voluntary running wheel activity [68]. However, we found no interaction between accesses to sucrose drinking and running wheels in *Gria1−/−* and WT mice on control diet, and unexpectedly, treatment with lithium and valproate rather increased than suppressed sucrose preference. Unlike sucrose consumption, which increased in both *Gria1−/−* and WT mice by valproate and lithium, running activity increased significantly only in WT animals. Rewarding responses were not consistently affected by the drugs in the present study.

The main effects of the drugs studied here were on hyperactivity of the *Gria1−/−* animals, but they produced also some effects on other behaviours. Chronic lithium reduced the frequency of contacts to new objects in the open field, as did valproate. Valproate induced an anxiolytic-like effect in the WT mice that was not observed in the *Gria1−/−* mice. In social interaction test, valproate prolonged reciprocal social contacts only in the *Gria1−/−* mice. The effects of lithium and valproate in different tests might be related to increased habituation. Topiramate had an anxiogenic-like effect in the *Gria1−/−* animals. Topiramate and lamotrigine were not studied here as widely as lithium and valproate for other behaviours than hyperactivity. Even though we did not find any significant difference in the behavior of mice differently housed (see the immobility times in FST *vs*. TST in the Results section), deprivation of social contacts is known to impair behavior [69]. Isolation of rats increases motivational value of sucrose [35] and number of social contacts [70], [71]. Multiple testing can also have a substantial influence on behavior [72]. In the present study, locomotor activity of mice with previous testing history was similar compared to those naïve to experimentation. However, this does not straightforwardly imply that the behavior observed in other tests in a multiple testing battery would not be affected by the prior testing and associated stress. Still, behavioural test batteries in rodents are widely used and favoured for wide behavioural screening and designed to cover many distinct behavioural domains relevant for human neuropsychiatric disorders [73]. We conclude that in the present experiments, the main effects that regulated the behaviour, especially the hyperactivity, were the mouse genotype and chronic drug treatments.

Rodent models in neuropsychiatry usually phenocopy only some aspects of a disease, such as hyperactivity. Hyperactivity is a shared feature among several psychiatric conditions, such as mania, anxiety, attention deficit hyperactivity disorder and autism spectrum disorders, in addition to some forms of schizophrenia. Animal models are valuable tools for studying predictive validity of treatments, but even with partial face validity, they usually have weak construct validity [74]. In *Gria1−/−* mouse line, hyperactivity is pronounced and concomitant with other behavioral abnormalities indicative of highly disinhibited behavior. Moreover, as we show here, their behavioral abnormalities and deficits are controlled by mood stabilizing medications. Importantly, AMPA receptors, as determinants of synaptic plasticity might have a critical role during late adolescence for the onset of cognitive and behavioral abnormalities for neuropsychiatric disorders. Ablation of GluA1 subunit from the hippocampus in late adolescence reproduced all behavioural abnormalities of mice with global deletion of GluA1 subunit, except for social deficits [75]. However, aggressiveness as a correlate of irritability and easily provoked behavior is lacking in *Gria1−/−* mice [29]. There are two other interesting glutamate-linked models for mania-like hyperactivity. They include the mice deficient in kainate receptor GluK2 subunit (*Grik2−/−*) [76], in which chronic treatment with lithium reduces hyperactivity, risk-taking behaviour and aggressive manic displays, and the *Shank3* overexpressing mice [77], in which acute valproate, but not chronic lithium, reduces hyperactivity. While SHANK proteins are postsynaptic density scaffolding proteins in glutamate synapses and implicated in a number of neuropsychiatric disorders [78], heteromeric kainate receptors, assembling also GluK2 subunits, regulate presynaptically and postsynaptically neurotransmission of both interneurons and principal neurons [79], [80]. Together with the present results on *Gria1−/−* mouse line, these models suggest an important role for excitatory glutamate transmission in disorders with hyperactivity.

In conclusion, this is the first report to describe behavioural effects of chronic treatments with different clinically used mood stabilizers in the AMPA receptor GluA1 subunit-deficient mice using test batteries specific to some hyperactive facets of bipolar illness, schizophrenia and schizoaffective disorder. *Gria1−/−* mice showed disinhibited risk-taking behaviour, hyperlocomotion and social deficits, which were at least partially reversed by mood stabilizers, and therefore, we suggest that this mouse line can be used as a model for screening for novel drugs to treat hyperactive neuropsychiatric disorders.

## Supporting Information

Table S1 The ARRIVE Guidelines Checklist.
(PDF)
